# *N*-Phenyl-2-(propan-2-yl­idene)­hydrazine­carboxamide

**DOI:** 10.1107/S1600536812004904

**Published:** 2012-02-10

**Authors:** Mohamed I. Attia, Hazem A. Ghabbour, Aida A. El-Azzouny, Ching Kheng Quah, Hoong-Kun Fun

**Affiliations:** a Department of Pharmaceutical Chemistry, College of Pharmacy, King Saud University, Riyadh 11451, Saudi Arabia; b Medicinal and Pharmaceutical Chemistry Department, Pharmaceutical and Drug Industries Research Division, National Research Centre, 12622, Dokki, Giza, Egypt; c X-ray Crystallography Unit, School of Physics, Universiti Sains Malaysia, 11800 USM, Penang, Malaysia

## Abstract

In the title compound, C_10_H_13_N_3_O, the hydrazinecarboxamide N—N—C(=O)—N unit is nearly planar [maximum deviation = 0.018 (2) Å] and is inclined at a dihedral angle of 8.45 (10)° with respect to the plane of the phenyl ring. The mol­ecular structure is stabilized by an intra­molecular C—H⋯O hydrogen bond which generates an *S*(6) ring motif. In the crystal, mol­ecules are linked into an inversion dimer by pairs of N—H⋯O and C—H⋯O hydrogen bonds.

## Related literature

For general background to and the pharmacological activities of the title compound, see: Sander & Shorvon (1987 ▶); Dimmock *et al.* (1993 ▶). For the preparation of the starting material of the title compound, see: Aboul-Enein *et al.* (2012 ▶). For standard bond-length data, see: Allen *et al.* (1987 ▶). For hydrogen-bond motifs, see: Bernstein *et al.* (1995 ▶). For a related compound, see: Thirumurugan *et al.* (2006 ▶).
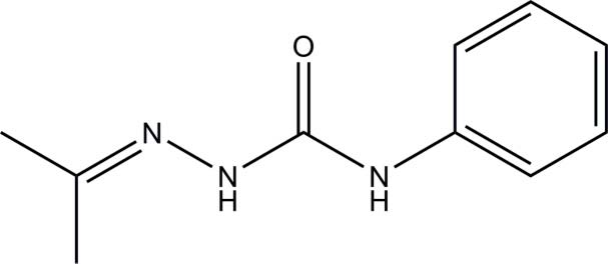


## Experimental

### 

#### Crystal data

C_10_H_13_N_3_O
*M* *_r_* = 191.23
Monoclinic, 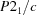

*a* = 6.2225 (3) Å
*b* = 15.3429 (7) Å
*c* = 11.8897 (5) Å
β = 112.283 (4)°
*V* = 1050.35 (8) Å^3^
*Z* = 4
Cu *K*α radiation
μ = 0.66 mm^−1^
*T* = 296 K
0.50 × 0.11 × 0.08 mm

#### Data collection

Bruker SMART APEXII CCD area-detector diffractometer
Absorption correction: multi-scan (*SADABS*; Bruker, 2009 ▶) *T*_min_ = 0.438, *T*_max_ = 0.949
7990 measured reflections
1657 independent reflections
938 reflections with *I* > 2σ(*I*)
*R*_int_ = 0.135

#### Refinement

*R*[*F* ^2^ > 2σ(*F* ^2^)] = 0.048
*wR*(*F* ^2^) = 0.142
*S* = 0.95
1657 reflections
130 parameters
H-atom parameters constrained
Δρ_max_ = 0.18 e Å^−3^
Δρ_min_ = −0.13 e Å^−3^

### 

Data collection: *APEX2* (Bruker, 2009 ▶); cell refinement: *SAINT* (Bruker, 2009 ▶); data reduction: *SAINT*; program(s) used to solve structure: *SHELXTL* (Sheldrick, 2008 ▶); program(s) used to refine structure: *SHELXTL*; molecular graphics: *SHELXTL*; software used to prepare material for publication: *SHELXTL* and *PLATON* (Spek, 2009 ▶).

## Supplementary Material

Crystal structure: contains datablock(s) global, I. DOI: 10.1107/S1600536812004904/is5068sup1.cif

Structure factors: contains datablock(s) I. DOI: 10.1107/S1600536812004904/is5068Isup2.hkl

Supplementary material file. DOI: 10.1107/S1600536812004904/is5068Isup3.cml

Additional supplementary materials:  crystallographic information; 3D view; checkCIF report

## Figures and Tables

**Table 1:** Hydrogen-bond geometry (Å, °)

*D*—H⋯*A*	*D*—H	H⋯*A*	*D*⋯*A*	*D*—H⋯*A*
N2—H2⋯O1^i^	0.87	2.04	2.892 (3)	168
C1—H1*A*⋯O1	0.93	2.29	2.879 (3)	120
C9—H9*A*⋯O1^i^	0.96	2.50	3.366 (3)	149

Symmetry code: (i) 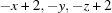.

**Fig. 1.**
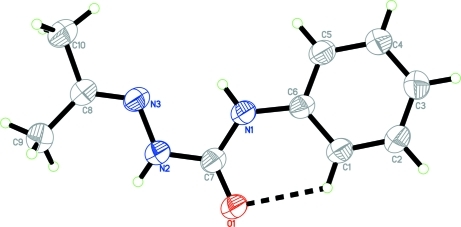
The molecular structure of the title compound showing 50% probability displacement ellipsoids for non-H atoms.

**Fig. 2.**
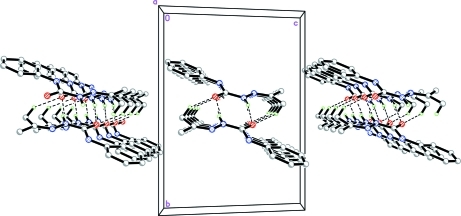
The crystal structure of the title compound, viewed along the a axis. H atoms not involved in hydrogen bonds (dashed lines) have been omitted for clarity.

## supplementary crystallographic information

### Comment

Epilepsy is one of the most widespread pathologies of the human brain, affecting approximately 1% of world population. Nevertheless, in the case of single drug treatment, the number of non-responding patients is as high as 30% and in chronic medication with currently available antiepileptic drugs (AEDs) may result in severe side-effects and undesired drug interactions (Sander & Shorvon, 1987). That is why, in recent years, intensive research has been carried out aiming at the development of new therapeutic strategies for epilepsy. Arylsemicarbazones have been documented to display significant anticonvulsant activity through the work of Dimmock and his colleagues (Dimmock *et al.*, 1993). Arylsemicarbazones are structurally dissimilar from many common monocyclic anticonvulsants which incorporate the dicarboxamide functionality, such as hydantoins and succinimides, which may contribute to toxic side effects. In general, semicarbazones have rapid onsets of action and one of the ways in which these compounds exerted their anticonvulsant activity is likely to be their interaction with the chloride channels.

In the title molecule, Fig. 1, the hydrazinecarboxamide moiety (N1–N3/O1/C7) is nearly planar with a maximum deviation of 0.018 (2) Å at atom N1, and is inclined at an angle of 8.45 (10)° with the phenyl ring (C1–C6). Bond lengths (Allen *et al.*, 1987) and angles are within normal ranges and are comparable to a related structure (Thirumurugan *et al.*, 2006). The molecular structure is stabilized by an intramolecular C1—H1A···O1 hydrogen bond (Table 1), which generates an *S*(6) ring motifs (Bernstein *et al.*, 1995). In the crystal (Fig. 2), molecules are linked into an inversion dimer by pairs of intermolecular N2—H2···O1 and C9—H9A···O1 hydrogen bonds (Table 1).

### Experimental

A solution of *N*-phenylhydrazinecarboxamide (0.1 g, 0.66 mmol) (Aboul-Enein *et al.*, 2012) and two drops of acetic acid in acetone (5 ml) was stirred at room temperature for 18 h. The solvent was evaporated under reduced pressure and the residue was recrystallized from ethanol to give the title compound. *M.p.* : 429-430 K.

### Refinement

N-bound H atoms were located in a difference Fourier map [N—H = 0.8488 and 0.8694 Å] and refined using a riding model, with *U*_iso_(H) = 1.2 *U*_eq_(N). The remaining hydrogen atoms were positioned geometrically [C—H = 0.93 or 0.96 Å] and were refined using a riding model, with *U*_iso_(H) = 1.2 or 1.5 *U*_eq_(C). A rotating group model was applied to the methyl groups.

### Crystal data

**Table:**

C_10_H_13_N_3_O	*F*(000) = 408
*M**_r_* = 191.23	*D*_x_ = 1.209 Mg m^−^^3^
Monoclinic, *P*2_1_/*c*	Cu *K*α radiation, λ = 1.54178 Å
Hall symbol: -P 2ybc	Cell parameters from 751 reflections
*a* = 6.2225 (3) Å	θ = 5.0–67.2°
*b* = 15.3429 (7) Å	µ = 0.66 mm^−^^1^
*c* = 11.8897 (5) Å	*T* = 296 K
β = 112.283 (4)°	Needel, colourless
*V* = 1050.35 (8) Å^3^	0.50 × 0.11 × 0.08 mm
*Z* = 4	

### Data collection

**Table:**

Bruker SMART APEXII CCD area-detector diffractometer	1657 independent reflections
Radiation source: fine-focus sealed tube	938 reflections with *I* > 2σ(*I*)
Graphite monochromator	*R*_int_ = 0.135
φ and ω scans	θ_max_ = 63.0°, θ_min_ = 5.0°
Absorption correction: multi-scan (*SADABS*; Bruker, 2009)	*h* = −5→6
*T*_min_ = 0.438, *T*_max_ = 0.949	*k* = −17→17
7990 measured reflections	*l* = −13→13

### Refinement

**Table:**

Refinement on *F*^2^	Secondary atom site location: difference Fourier map
Least-squares matrix: full	Hydrogen site location: inferred from neighbouring sites
*R*[*F*^2^ > 2σ(*F*^2^)] = 0.048	H-atom parameters constrained
*wR*(*F*^2^) = 0.142	*w* = 1/[σ^2^(*F*_o_^2^) + (0.0676*P*)^2^] where *P* = (*F*_o_^2^ + 2*F*_c_^2^)/3
*S* = 0.95	(Δ/σ)_max_ = 0.002
1657 reflections	Δρ_max_ = 0.18 e Å^−^^3^
130 parameters	Δρ_min_ = −0.13 e Å^−^^3^
0 restraints	Extinction correction: *SHELXTL* (Sheldrick, 2008), Fc^*^=kFc[1+0.001xFc^2^λ^3^/sin(2θ)]^-1/4^
Primary atom site location: structure-invariant direct methods	Extinction coefficient: 0.0093 (12)

### Special details

**Table:**

Geometry. All esds (except the esd in the dihedral angle between two l.s. planes) are estimated using the full covariance matrix. The cell esds are taken into account individually in the estimation of esds in distances, angles and torsion angles; correlations between esds in cell parameters are only used when they are defined by crystal symmetry. An approximate (isotropic) treatment of cell esds is used for estimating esds involving l.s. planes.
Refinement. Refinement of F^2^ against ALL reflections. The weighted R-factor wR and goodness of fit S are based on F^2^, conventional R-factors R are based on F, with F set to zero for negative F^2^. The threshold expression of F^2^ > 2sigma(F^2^) is used only for calculating R-factors(gt) etc. and is not relevant to the choice of reflections for refinement. R-factors based on F^2^ are statistically about twice as large as those based on F, and R- factors based on ALL data will be even larger.

### Fractional atomic coordinates and isotropic or equivalent isotropic displacement parameters (Å^2^)

**Table:**

	*x*	*y*	*z*	*U*_iso_*/*U*_eq_	
O1	0.8269 (3)	0.06411 (10)	0.88218 (14)	0.0795 (5)	
N1	0.5175 (3)	0.12926 (11)	0.90676 (16)	0.0686 (6)	
H1	0.4585	0.1299	0.9605	0.082*	
N2	0.7965 (3)	0.05943 (12)	1.06500 (16)	0.0691 (6)	
H2	0.9184	0.0260	1.0912	0.083*	
N3	0.6658 (3)	0.08137 (11)	1.13237 (17)	0.0671 (5)	
C1	0.4742 (4)	0.16254 (14)	0.6968 (2)	0.0740 (7)	
H1A	0.6202	0.1402	0.7087	0.089*	
C2	0.3356 (5)	0.19777 (16)	0.5857 (2)	0.0844 (7)	
H2A	0.3905	0.1989	0.5230	0.101*	
C3	0.1202 (4)	0.23094 (16)	0.5654 (2)	0.0855 (8)	
H3A	0.0301	0.2543	0.4900	0.103*	
C4	0.0386 (4)	0.22947 (15)	0.6575 (2)	0.0800 (7)	
H4A	−0.1074	0.2521	0.6450	0.096*	
C5	0.1727 (4)	0.19460 (13)	0.7679 (2)	0.0715 (7)	
H5A	0.1154	0.1933	0.8296	0.086*	
C6	0.3922 (4)	0.16118 (12)	0.78967 (19)	0.0602 (6)	
C7	0.7190 (4)	0.08280 (14)	0.9466 (2)	0.0640 (6)	
C8	0.7504 (4)	0.06860 (13)	1.2464 (2)	0.0669 (6)	
C9	0.9849 (4)	0.03171 (16)	1.3183 (2)	0.0873 (8)	
H9A	1.0181	−0.0148	1.2733	0.131*	
H9B	0.9871	0.0097	1.3943	0.131*	
H9C	1.1001	0.0765	1.3333	0.131*	
C10	0.6029 (4)	0.09321 (16)	1.3149 (2)	0.0883 (8)	
H10A	0.4583	0.1166	1.2596	0.132*	
H10B	0.6818	0.1364	1.3748	0.132*	
H10C	0.5735	0.0426	1.3543	0.132*	

### Atomic displacement parameters (Å^2^)

**Table:**

	*U*^11^	*U*^22^	*U*^33^	*U*^12^	*U*^13^	*U*^23^
O1	0.0855 (11)	0.0935 (11)	0.0727 (11)	0.0206 (8)	0.0449 (9)	0.0105 (9)
N1	0.0766 (12)	0.0733 (11)	0.0652 (11)	0.0133 (10)	0.0374 (9)	0.0071 (10)
N2	0.0719 (12)	0.0784 (12)	0.0636 (12)	0.0100 (9)	0.0331 (9)	0.0026 (10)
N3	0.0725 (13)	0.0741 (11)	0.0650 (12)	0.0015 (9)	0.0375 (9)	−0.0009 (10)
C1	0.0778 (16)	0.0797 (14)	0.0764 (16)	0.0085 (11)	0.0427 (12)	0.0103 (13)
C2	0.0960 (19)	0.0941 (16)	0.0774 (18)	0.0090 (15)	0.0490 (13)	0.0165 (14)
C3	0.0818 (18)	0.0957 (17)	0.0816 (18)	0.0115 (14)	0.0338 (13)	0.0227 (15)
C4	0.0774 (17)	0.0878 (16)	0.0817 (18)	0.0106 (12)	0.0379 (13)	0.0125 (15)
C5	0.0796 (16)	0.0728 (13)	0.0733 (16)	0.0049 (12)	0.0415 (12)	0.0028 (12)
C6	0.0688 (15)	0.0544 (11)	0.0658 (14)	0.0002 (10)	0.0351 (10)	−0.0015 (11)
C7	0.0725 (16)	0.0627 (12)	0.0636 (16)	0.0014 (11)	0.0333 (11)	−0.0006 (12)
C8	0.0744 (16)	0.0662 (11)	0.0662 (16)	−0.0066 (11)	0.0335 (12)	−0.0052 (12)
C9	0.0894 (17)	0.0979 (16)	0.0719 (15)	0.0069 (13)	0.0275 (13)	−0.0016 (14)
C10	0.0961 (19)	0.1029 (17)	0.0802 (17)	0.0004 (15)	0.0496 (14)	−0.0007 (15)

### Geometric parameters (Å, º)

**Table:**

O1—C7	1.229 (3)	C3—H3A	0.9300
N1—C7	1.362 (3)	C4—C5	1.369 (3)
N1—C6	1.401 (2)	C4—H4A	0.9300
N1—H1	0.8488	C5—C6	1.388 (3)
N2—C7	1.352 (3)	C5—H5A	0.9300
N2—N3	1.382 (2)	C8—C10	1.487 (3)
N2—H2	0.8694	C8—C9	1.495 (3)
N3—C8	1.270 (2)	C9—H9A	0.9600
C1—C6	1.381 (3)	C9—H9B	0.9600
C1—C2	1.385 (3)	C9—H9C	0.9600
C1—H1A	0.9300	C10—H10A	0.9600
C2—C3	1.367 (3)	C10—H10B	0.9600
C2—H2A	0.9300	C10—H10C	0.9600
C3—C4	1.371 (3)		
			
C7—N1—C6	128.38 (18)	C1—C6—C5	118.7 (2)
C7—N1—H1	110.4	C1—C6—N1	124.4 (2)
C6—N1—H1	120.5	C5—C6—N1	116.88 (18)
C7—N2—N3	118.88 (18)	O1—C7—N2	121.6 (2)
C7—N2—H2	116.6	O1—C7—N1	123.6 (2)
N3—N2—H2	124.1	N2—C7—N1	114.8 (2)
C8—N3—N2	118.99 (18)	N3—C8—C10	117.0 (2)
C6—C1—C2	118.9 (2)	N3—C8—C9	126.0 (2)
C6—C1—H1A	120.6	C10—C8—C9	117.0 (2)
C2—C1—H1A	120.6	C8—C9—H9A	109.5
C3—C2—C1	121.9 (2)	C8—C9—H9B	109.5
C3—C2—H2A	119.1	H9A—C9—H9B	109.5
C1—C2—H2A	119.1	C8—C9—H9C	109.5
C2—C3—C4	119.3 (2)	H9A—C9—H9C	109.5
C2—C3—H3A	120.4	H9B—C9—H9C	109.5
C4—C3—H3A	120.4	C8—C10—H10A	109.5
C5—C4—C3	119.7 (2)	C8—C10—H10B	109.5
C5—C4—H4A	120.1	H10A—C10—H10B	109.5
C3—C4—H4A	120.1	C8—C10—H10C	109.5
C4—C5—C6	121.5 (2)	H10A—C10—H10C	109.5
C4—C5—H5A	119.3	H10B—C10—H10C	109.5
C6—C5—H5A	119.3		
			
C7—N2—N3—C8	−171.93 (18)	C7—N1—C6—C1	−11.0 (3)
C6—C1—C2—C3	−0.1 (4)	C7—N1—C6—C5	169.99 (19)
C1—C2—C3—C4	0.0 (4)	N3—N2—C7—O1	−179.16 (18)
C2—C3—C4—C5	−0.3 (4)	N3—N2—C7—N1	2.0 (3)
C3—C4—C5—C6	0.7 (3)	C6—N1—C7—O1	2.5 (3)
C2—C1—C6—C5	0.5 (3)	C6—N1—C7—N2	−178.70 (18)
C2—C1—C6—N1	−178.47 (19)	N2—N3—C8—C10	−179.96 (18)
C4—C5—C6—C1	−0.8 (3)	N2—N3—C8—C9	0.5 (3)
C4—C5—C6—N1	178.26 (18)		

### Hydrogen-bond geometry (Å, º)

**Table:**

*D*—H···*A*	*D*—H	H···*A*	*D*···*A*	*D*—H···*A*
N2—H2···O1^i^	0.87	2.04	2.892 (3)	168
C1—H1*A*···O1	0.93	2.29	2.879 (3)	120
C9—H9*A*···O1^i^	0.96	2.50	3.366 (3)	149

Symmetry code: (i) −*x*+2, −*y*, −*z*+2.
